# MicroRNA Expression Changes during Interferon-Beta Treatment in the Peripheral Blood of Multiple Sclerosis Patients

**DOI:** 10.3390/ijms140816087

**Published:** 2013-08-05

**Authors:** Michael Hecker, Madhan Thamilarasan, Dirk Koczan, Ina Schröder, Kristin Flechtner, Sherry Freiesleben, Georg Füllen, Hans-Jürgen Thiesen, Uwe Klaus Zettl

**Affiliations:** 1 Steinbeis Transfer Center for Proteome Analysis, Schillingallee 68, 18057 Rostock, Germany; 2 Department of Neurology, Division of Neuroimmunology, University of Rostock, Gehlsheimer Str. 20, 18147 Rostock, Germany; E-Mails: madhangp@gmail.com (M.T.); ina.schroeder@med.uni-rostock.de (I.S.); uwe.zettl@med.uni-rostock.de (U.K.Z.); 3 Institute of Immunology, University of Rostock, Schillingallee 70, 18055 Rostock, Germany; E-Mails: dirk.koczan@med.uni-rostock.de (D.K.); kristin.flechtner@googlemail.com (K.F.); hj.thiesen@gmx.de (H.-J.T.); 4 Institute for Biostatistics and Informatics in Medicine and Ageing Research, University of Rostock, Ernst-Heydemann-Str. 8, 18057 Rostock, Germany; E-Mails: sherry.freiesleben@uni-rostock.de (S.F.); fuellen@uni-rostock.de (G.F.)

**Keywords:** interferon-beta, multiple sclerosis, peripheral blood, microRNA, gene expression

## Abstract

MicroRNAs (miRNAs) are small non-coding RNA molecules acting as post-transcriptional regulators of gene expression. They are involved in many biological processes, and their dysregulation is implicated in various diseases, including multiple sclerosis (MS). Interferon-beta (IFN-beta) is widely used as a first-line immunomodulatory treatment of MS patients. Here, we present the first longitudinal study on the miRNA expression changes in response to IFN-beta therapy. Peripheral blood mononuclear cells (PBMC) were obtained before treatment initiation as well as after two days, four days, and one month, from patients with clinically isolated syndrome (CIS) and patients with relapsing-remitting MS (RRMS). We measured the expression of 651 mature miRNAs and about 19,000 mRNAs in parallel using real-time PCR arrays and Affymetrix microarrays. We observed that the up-regulation of IFN-beta-responsive genes is accompanied by a down-regulation of several miRNAs, including members of the mir-29 family. These differentially expressed miRNAs were found to be associated with apoptotic processes and IFN feedback loops. A network of miRNA-mRNA target interactions was constructed by integrating the information from different databases. Our results suggest that miRNA-mediated regulation plays an important role in the mechanisms of action of IFN-beta, not only in the treatment of MS but also in normal immune responses. miRNA expression levels in the blood may serve as a biomarker of the biological effects of IFN-beta therapy that may predict individual disease activity and progression.

## 1. Introduction

Multiple sclerosis (MS) is a chronic disease of the central nervous system (CNS), which is characterized by multiple discrete areas of inflammatory demyelination, axonal degeneration, and glial scarring. The resulting loss of neurons and axons leads to diverse neurological symptoms, progressive disability, and a significant decrease in quality of life. The disease usually begins in early adulthood, and is more common in females. Different types of MS are distinguished: In about 85% of patients, the disease starts with a single demyelinating episode (clinically isolated syndrome, CIS) and progresses to a relapsing-remitting course (RRMS) with acute exacerbations and periods of remission [1–4].

A number of disease-modifying therapies for MS are available, and they are especially effective when applied in the early stages of the disease [4,5]. Injections of recombinant interferon-beta (IFN-beta) are considered a first-line option in the treatment of RRMS. IFN-beta has been shown to reduce the number of relapses and to suppress the accumulation of new inflammatory lesions in the brain. Three different preparations of IFN-beta are in clinical use. They differ in dose, route, and frequency of IFN-beta administration, but they are comparable regarding clinical efficacy [6].

IFN-beta has broad effects on the gene regulation of blood cells [7–11]. This has been shown by several studies that used microarray technology to analyze the gene expression dynamics in the peripheral blood of MS patients in response to IFN-beta therapy. In this way, more than a hundred genes have consistently been found differentially expressed during treatment [10]. The transcript levels of most of these genes are up-regulated within a few hours after IFN-beta injection, and they return to pre-treatment levels after a few days [12,13]. These IFN-beta-responsive genes are believed to mediate the beneficial effects of the treatment through immunomodulatory, antiproliferative, and antipathogenic processes [7–9].

While the therapeutic effects on the regulation of mRNAs have been extensively investigated, studies on the regulation of microRNAs (miRNAs) are lacking. miRNAs are a distinct class of small (~22 nt) non-coding RNA molecules [14]. They originate from precursor RNAs (pre-miRNAs) found in longer primary transcripts (pri-miRNAs), which often also contain the exons of an mRNA. Mature miRNAs act as post-transcriptional regulators. They repress gene expression via base-pairing with complementary sequences within the 3′ untranslated regions (UTRs) of target mRNAs. This interaction results in gene silencing by translational repression or target degradation. A miRNA can have hundreds of different mRNA targets, and a target might be regulated by a combination of multiple miRNAs [15]. The human genome encodes over 1000 miRNAs [16,17]. miRNAs are thus likely involved in most biological processes, and they play essential roles in the immune system and in the correct function of the CNS [18,19].

Dysregulated expression of miRNAs is associated with pathological conditions, including neurological diseases. Human MS studies showed altered miRNA expression in peripheral blood samples, lymphocyte subpopulations, and active CNS lesions from MS patients [20–22]. Studies with the animal model of MS, experimental autoimmune encephalomyelitis (EAE), also support the involvement of miRNAs in this disease [23,24]. These findings provided important insights into the pathophysiology of MS and opened a new avenue in biomarker research. If miRNA levels in the blood or brain of MS patients correlate with disease stage and progression of disability, they may also support early diagnosis and effective treatment in future [25].

In order to better understand the molecular mechanisms of action of IFN-beta therapy, it is important to investigate the miRNA expression dynamics during therapy. The regulation of miRNAs may contribute to the immunomodulatory and clinical effects of the treatment. Moreover, miRNAs might be markers for characterizing the biological response to IFN-beta. miRNA biomarkers for treatment monitoring could be useful in the individual management of disease activity. However, so far, there is only one study on the expression of miRNAs in MS patients during IFN-beta therapy: Waschbisch *et al.* obtained peripheral blood mononuclear cells (PBMC) from patients with RRMS, and analyzed the expression of five selected miRNAs by real-time PCR [26]. They compared the miRNA levels between treatment-naive patients (*n* = 36), IFN-beta-treated patients (*n* = 18), and patients treated with glatiramer acetate (GA, *n* = 20). As a result, none of the five miRNAs was differentially expressed in IFN-beta-treated patients, but *hsa-miR-146a-5p* and *hsa-miR-142-3p* were expressed at significantly lower levels in GA-treated patients [26]. Other researchers used microarrays to study the expression of hundreds of miRNAs in IFN-stimulated cells. In this way, O’Connell *et al.* observed that *hsa-miR-155-5p* is induced in primary murine macrophages after exposure to IFN-beta for 6 h [27]. Pedersen *et al.* studied the regulation of miRNAs in Huh7 cells and primary hepatocytes, which were stimulated with different concentrations of IFN-beta for up to 48 h [28]. They observed increased and reduced miRNA expression in response to IFN-beta, and showed that some of the IFN-beta-induced miRNAs mediate antiviral effects against hepatitis C virus. This provides an example of miRNAs as components of the innate immune response.

In this study, we used microarrays to investigate in parallel the expression dynamics of mRNAs and miRNAs in PBMC of patients with CIS or RRMS in response to therapy with subcutaneous (sc.) IFN-beta. The blood samples were obtained longitudinally from six patients at four time points in the early phase of therapy, namely before the first (baseline), second, and third IFN-beta injection as well as after one month of treatment. We then screened for significant changes in miRNA and mRNA expression, and identified several miRNAs as differentially expressed during therapy. Information of different databases was then integrated [22,29] to examine whether the expression of these miRNAs is cell type-specific and correlates with the levels of their target mRNAs. Predicted and experimentally verified miRNA-mRNA interactions were compiled to construct a network of IFN-beta-responsive genes and miRNAs. To our knowledge, this is the first genome-wide miRNA profiling study on the *in vivo* effects of IFN-beta treatment in MS.

## 2. Results and Discussion

### 2.1. Study Population

Six female patients of Western European descent, and diagnosed with CIS (*n* = 2) or RRMS (*n* = 4), were recruited for this study (Pat1-6, mean age 37.5 years, Table 1). The patients were treatment-naive and started an immunomodulatory therapy with IFN-beta-1b (Betaferon, Bayer HealthCare) administered subcutaneously every other day. In the first weeks, the Betaferon titration pack was used, hence the patients started with a low dose (62.5 μg for the first three injections) that was gradually increased to the full dose (250 μg) after three weeks. All patients were continuously treated with IFN-beta-1b for at least one year. During follow-up, they were monitored for relapses and rated using the Expanded Disability Status Scale (EDSS). The individual disease activity during therapy was relatively low: Four of the patients (Pat1-4) were relapse-free and showed no disability progression within the first year of treatment (Table 1). The two patients with CIS (Pat1 and Pat5) did not convert to clinically definite MS in this period of time.

Note that the patient group included only women. A differential hormonal regulation of immune system genes in blood cells has been described for different phases of the menstrual cycle [30]. Such differences in gene expression may have led to increased variance in the data. However, prior mRNA profiling studies observed no significant gender-specific differences in the gene expression signature in response to IFN-beta therapy [8,31], and this seems to be the case regarding the expression of miRNAs as well (see Section 2.5).

### 2.2. Parallel Measurement of mRNAs and MicroRNAs in Blood Cells

Patient blood samples were drawn immediately before first IFN-beta injection as well as two days, four days, and one month post therapy initiation. Total RNA of Ficoll-isolated PBMC from each sample was extracted to measure the levels of mRNAs and miRNAs with different platforms. We used TaqMan Array Human MicroRNA cards (Applied Biosystems, Foster City, CA, USA) to quantify the expression of 651 mature miRNAs and Affymetrix HG-U133 Plus 2.0 microarrays (Affymetrix, Santa Clara, CA, USA) to quantify the expression of about 19,000 mRNAs. In this way, we obtained in parallel the mRNA and miRNA expression profiles from six patients (Pat1-6) within the first month of IFN-beta treatment.

The data were preprocessed as described in Section 3.6. Relatively low variation in the transcriptome profiles indicated high data quality, comparable to our previous microarray time course data sets [8,31,32]. In the miRNA data, systematic and stochastic variation was higher. For the TaqMan miRNA B-cards of Pat5, the raw threshold cycle (Ct) values were generally higher (Supplementary File 1) due to an unknown measurement bias. In the PBMC samples of the other five patients, approximately 400 miRNAs could be detected (Ct < 38) with the TaqMan miRNA arrays (Table 2). The raw TaqMan data were transformed to the linear scale, and coefficients of variation (*CV*) were calculated to assess the effects of data normalization. The normalization decreased the average *CV* over all 768 measured assays from 0.953 to 0.894. The *CV* for the housekeeping miRNA *hsa-miR-191-5p* [33] was 0.183. In comparison, assays for the non-coding RNAs *U6*, *U44* and *U48* had *CV*s of >0.35. The *CV* for the housekeeping mRNA GAPDH was 0.071.

### 2.3. Analysis of mRNA Expression Dynamics

**Figure 1:**
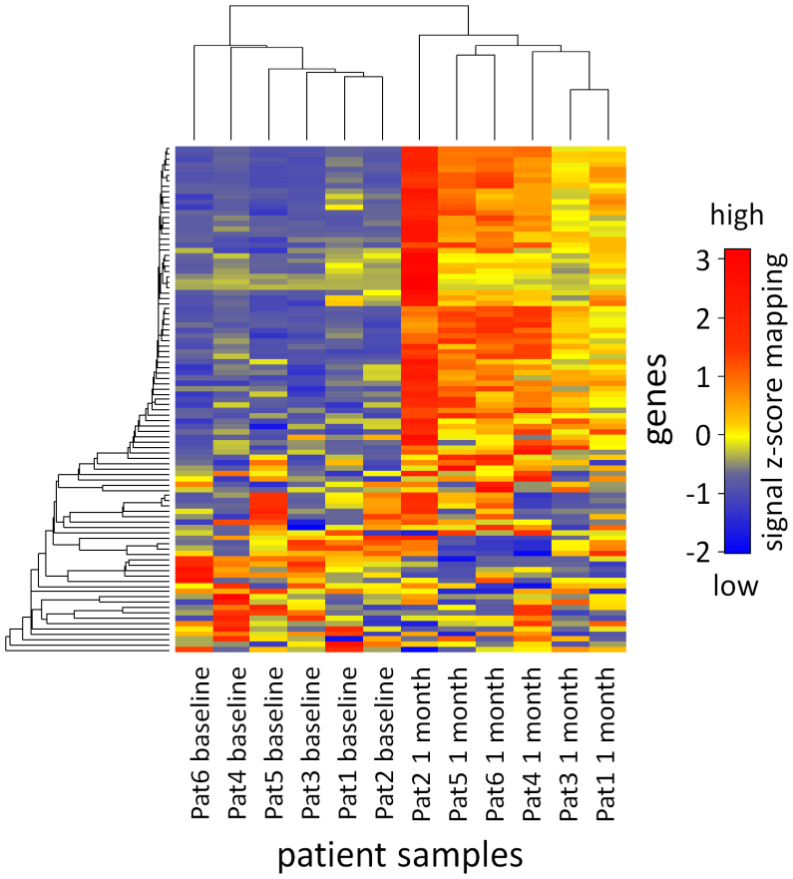
Heat map visualization of the mRNA expression changes in response to IFN-beta. Shown are the baseline and one month transcript levels of the 95 genes that were identified as differentially expressed during IFN-beta therapy. The patient samples are represented in the columns, the genes are represented in the rows, and the gene expression levels were centered and scaled in row direction (z-scores). The clustering analysis clearly separated the PBMC samples obtained at baseline and after one month of therapy. The row labels of the heat map (*i.e.*, the respective genes) are given in Supplementary File 2. The upper half of the heat map contains most of the IFN-beta-induced genes.

We filtered for mRNAs showing strong expression changes in response to IFN-beta therapy by comparing the baseline expression levels with the expression levels at the three time points during treatment. The MAID filtering method [34] was used to analyze the mRNA dynamics. As a result, 14, 34, and 66 genes were found to be expressed at higher or lower levels after two days, four days, and one month, respectively. In total, 95 genes were identified as up-regulated (*n* = 75) or down-regulated (*n* = 20) in the early course of the therapy (Supplementary File 2). The gene expression changes in the first month of subcutaneous IFN-beta-1b treatment are visualized in the heat map in Figure 1.

A permutation test (see Section 3.7) revealed that the number of 95 differentially expressed genes is significantly higher than would be expected by chance. In randomly permuted data sets, 29.6 genes on average were filtered. The number of filtered genes was below 95 in 98.8% of the permutations, which implies an empirical *p*-value of <0.05, demonstrating that most of the mRNA expression changes that we found are indeed due to the therapy.

Despite the relatively small patient cohort (*n* = 6), the mRNA results were quite consistent with the literature. In another study, we already analyzed the PBMC gene expression profiles of a larger group of MS patients (*n* = 25) treated with IFN-beta-1b sc. [31]. In total, 63 of the 95 differentially expressed genes were also filtered in the previous study. Recently, we completed a similar microarray study on the effects of IFN-beta-1a sc. [8], in which 49 of the 95 genes were already identified as transcriptionally modulated (Supplementary File 2). Moreover, more up-regulated than down-regulated genes were filtered, which also confirms previous findings [10]. After the first IFN-beta injections, fewer genes were altered in expression than after one month (*cf.* Goertsches *et al.*, 2010 [31]). This can be explained by the fact that the patients started the first week of the therapy with a quarter of the full dose (Betaferon titration pack). Most of the filtered genes are part of an up-regulated type I IFN signature. For instance, *IFI6*, *IFI44L*, and *SIGLEC1* are known type I IFN-induced genes, which were up-regulated at all time points during therapy in comparison to baseline. In contrast, FCER1A was consistently down-regulated in response to therapy as has been described previously as well [8,31,32]. Regarding the functions of these genes, the reader is referred to the literature [7–9].

### 2.4. Analysis of MicroRNA Expression Dynamics

**Figure 2:**
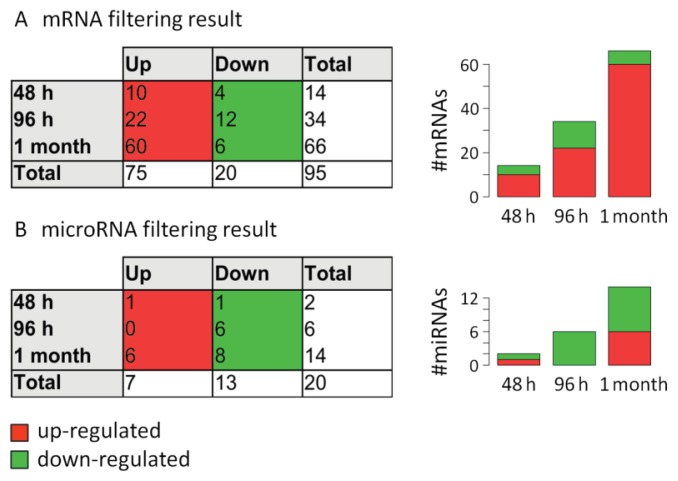
Summary of the filtering of IFN-beta-responsive mRNAs and microRNAs. PBMC expression levels during therapy were compared to pre-treatment levels. The number (#) of differentially expressed mRNAs and miRNAs is depicted in the tables and bar plots. The row “Total” gives the union set over all three time point comparisons. (**A**) In the mRNA data, 95 genes were found to be modulated in expression in response to IFN-beta-1b treatment. As expected, most of them were up-regulated (*n* = 75) and known type I IFN-induced genes, and the strongest changes were observed at one month versus baseline; (**B**) In the miRNA data, the filtering method identified more down-regulated than up-regulated miRNAs during therapy, again with the strongest effects seen after one month.

As for the mRNA data, the MAID filtering method [34] was used to identify miRNAs that are differentially expressed in PBMC within the first month of IFN-beta treatment. When we compared the expression levels at the three time points during therapy with the expression levels at baseline, 20 different miRNAs were filtered. Of these, seven miRNAs appeared as up-regulated and 13 miRNAs appeared as down-regulated in response to the therapy (Figure 2, Table 3 and Supplementary File 3). According to the permutation test, this is significantly more than expected by chance: In only 0.6% of the randomly permuted data sets, 20 (or more) miRNAs were filtered, and only 7.7 miRNAs were filtered on average.

Two of the 20 miRNAs (*hsa-miR-149-5p* and *hsa-miR-708-5p*) were filtered at two different time points. For the remaining miRNAs, the expression changes were not very stable in the course of therapy. This may be due to the small number of patients and the fact that the accuracy of miRNA measurements is in general limited. Therefore, our list of 20 miRNAs represents candidates that have to be validated in a larger patient cohort using, e.g., single real-time PCR assays.

Most of the miRNAs (*n* = 14) were filtered as up-regulated or down-regulated one month after IFN-beta-1b sc. treatment initiation. This corresponds to the results of the gene expression profiling, where the strongest changes in mRNA levels were also observed after one month (Figure 2). Previous studies demonstrated that the majority of IFN-beta-responsive genes can be seen at this time point [8,31]. Therefore, we hypothesize that, similarly, the number of miRNAs that are modulated in expression is not much higher after long-term treatment. Instead, the development of neutralizing antibodies (NAb) to IFN-beta might impair the biological response to the drug in some patients [35]. However, further studies are needed to investigate the long-term regulation of miRNAs and the potential effects of NAb.

Apparently, there are more down-regulated than up-regulated miRNAs during therapy, which is the opposite of the mRNA results. This suggests that the induction of IFN-beta-responsive genes is paralleled by a preferential down-regulation of miRNAs, which is plausible given that miRNAs act as gene silencers. Therefore, we analyzed whether the miRNAs indeed participate in the regulation of the mRNA transcripts (see Section 2.7).

### 2.5. Validation of IFN-beta-Induced MicroRNA Expression Changes

We used Affymetrix miRNA microarrays to replicate the miRNA measurements of the PBMC samples from three patients (Pat1-3) before the start of IFN-beta therapy as well as after one month (see Section 3.4). These microarrays had a lower measurement range than the TaqMan miRNA arrays, though the comparability of the data was acceptable (Spearman’s rho = 0.823). The statistical analysis of this additional data set is limited by the small number of patients. However, when we compared the mean miRNA expression levels before and one month after treatment initiation, 13 of the 20 filtered miRNAs showed the same trend of up-regulation or down-regulation (Supplementary File 3). At the significance threshold alpha = 0.10, four miRNAs (*hsa-miR-29a-3p*, *hsa-miR-29c-3p*, *hsa-miR-193a-3p*, and *hsa-miR-532-5p*) were confirmed to be down-regulated during treatment.

**Figure 3:**
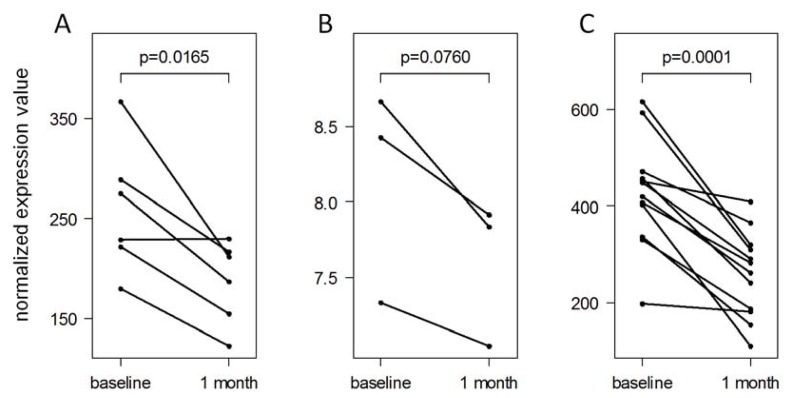
Down-regulation of *hsa-miR-29c-3p* in response to IFN-beta therapy. The *hsa-miR-29c-3p* expression dynamics within the first month of IFN-beta treatment are presented. (**A**) TaqMan miRNA cards revealed reduced levels of this miRNA in the PBMC of 6 patients (Pat1-6, the main cohort); (**B**) Affymetrix miRNA arrays were then used to replicate the measurement for three of these patients (Pat1-3); (**C**) Finally, the down-regulation of *hsa-miR-29c-3p* was confirmed in an independent group of 12 patients (the validation cohort) using TaqMan single-tube assays. The Affymetrix analysis was based on hybridization of miRNA molecules to probes (probe set “*hsa-miR-29c_st*”), whereas the TaqMan analyses were based on real-time PCR. The TaqMan data are in linear scale, and the Affymetrix data are in log2 scale due to a different data preprocessing.

For further validation, we selected five of the 20 filtered miRNAs to quantify their expression in PBMC of an independent cohort of 12 patients using TaqMan single-tube assays (Supplementary File 4 and Supplementary File 5). These 12 patients (8 RRMS/4 CIS, 7 females/5 males, mean age 36.2 years) also started a therapy with IFN-beta-1b sc. The PBMC were obtained again in a longitudinal manner before the first drug injection and after one month of treatment. In this data set, *hsa-miR-29a-3p* and *hsa-miR-29c-3p* could be confirmed as differentially expressed in response to IFN-beta therapy (*t*-test *p*-values < 0.001). *hsa-miR-29c-3p* was expressed at lower levels during therapy in comparison to pre-treatment levels in all 12 patients, hence independent of disease stage (CIS or RRMS), age, and gender (Figure 3). Additionally, *hsa-miR-532-5p* was confirmed to be down-regulated (*p*-value = 0.048). *hsa-miR-16-5p* and *hsa-miR-149-5p* showed the same trend of expression change as in the other data sets (TaqMan miRNA arrays and Affymetrix miRNA arrays), but this was not statistically significant (*p*-values > 0.10, Supplementary File 3).

### 2.6. Functions of the MicroRNAs in the Context of Multiple Sclerosis

The filtered miRNAs (Table 3) affect diverse cellular functions and pathways, and some of them have been implicated in MS. In our data set, *hsa-let-7a-5p* and *hsa-let-7b-5p*, which belong to the let-7 family, were expressed at higher levels during therapy. Lehmann *et al.* showed that let-7 family members can activate TLR7 signaling in macrophages and microglia, thereby inducing neurodegeneration [36]. In CD4+ T cells, let-7 miRNAs reduce the expression of IL10, a cytokine with anti-inflammatory properties [37]. The up-regulation of *hsa-let-7b-5p* by IFN-beta has already been demonstrated *in vitro* in primary macrophages [38]. Interestingly, *hsa-let-7b-5p* is in turn capable of binding the endogenous IFN-beta transcript, forming a negative feedback loop for the regulation of IFN-beta protein [38]. Recently, Gandhi *et al.* found altered *hsa-let-7a-5p* levels in the blood plasma of patients with the secondary-progressive form of MS, but not of patients with RRMS [39].

The observed up-regulation of *hsa-miR-16-5p* in response to IFN-beta therapy may restore the aberrant expression of this miRNA in the disease. Two studies observed reduced levels of *hsa-miR-16-5p* in the blood of RRMS patients. One study compared the expression in PBMC and CD4+ T cells from untreated patients and healthy donors [40]. The other study showed that *hsa-miR-16-5p* is down-regulated in B cells as well [41].

Three miRNAs of the mir-29 family were down-regulated one month after the start of therapy, and this was confirmed for two of them in an independent validation cohort of 12 patients (Figure 3). *hsa-miR-29a-3p* and *hsa-miR-29c-3p* were both expressed at relatively high levels (Supplementary File 3), and their mature sequences differ only in one base (Table 3). Therefore, it is likely that they play similar roles as post-transcriptional regulators. *hsa-miR-29a-3p* and *hsa-miR-29b-1-5p* belong to the same genomic cluster. Smith *et al.* demonstrated that IFN-gamma enhances the transcription of this *miR-29ab1* cluster, which acts in a negative feedback loop by regulating TBET and IFN-gamma [42]. Additionally, they showed decreased *hsa-miR-29b-3p* levels upon T cell activation in MS patients. This suggests a dysregulation of the feedback loop, which may bias T helper type 1 cell differentiation and may contribute to chronic inflammation [42]. Other studies also provided evidence that the members of the mir-29 family control innate and adaptive immune responses by targeting IFN-gamma [43,44]. The therapeutic down-modulation of mir-29 miRNAs might be mediated by NF-kappaB. The activation of NF-kappaB signaling, via ligation of Toll-like receptors, was shown to inhibit *miR-29ab1* expression [45]. Functionally, mir-29 promotes apoptosis, whereas repression of mir-29 levels is protective [45]. *hsa-miR-29a-3p* has been further shown to regulate myelin gene expression by Schwann cells [46].

*hsa-miR-181c-3p* was filtered as down-regulated during IFN-beta therapy. Several studies described the other strand of its pre-miRNA to be dysregulated in MS. Lower levels of *hsa-miR-181c-5p* were measured in PBMC [47] and in MS lesions [48] of patients in comparison to controls. On the other hand, *hsa-miR-181c-5p* seems to be overabundant in the cerebrospinal fluid of patients with MS [49]. However, the biological processes that are influenced by the *hsa-mir-181c* miRNAs and their role in MS therapy remain largely unknown.

The expression of the mir-193 family members *hsa-miR-193a-3p* and *hsa-miR-193a-5p* was repressed during the therapy (Table 3). A study by Lindberg *et al.* demonstrated increased expression of *hsa-miR-193a-5p* in CD4+ T cells of RRMS patients compared to healthy subjects [50]. Otaegui *et al.* confirmed the potential relevance of this miRNA duplex in MS. Based on a co-expression network analysis, they postulated that *hsa-miR-193a-3p* is related to the remission stage of MS [51]. Moreover, the precursor molecule *hsa-mir-193a* was found to modulate apoptotic processes by promoting CASP3 activation induced by TNFSF10 signaling [52]. TNFSF10 (=TRAIL), in turn, is a known IFN-beta-induced gene and was transcriptionally up-regulated in the patients’ PBMC (Supplementary File 2). The concomitant regulation of mir-193 miRNAs may thus contribute to the molecular mechanisms of action of IFN-beta.

Another miRNA caught our attention: *hsa-miR-223-3p* appeared to be the highest expressed miRNA in most PBMC samples (mean raw Ct value = 13.6). In a microarray study by Keller *et al.*, elevated levels of this miRNA were measured in the peripheral blood of RRMS patients as compared with healthy controls [53]. A significantly increased expression of *hsa-miR-223-3p* was later confirmed in PBMC from RRMS patients using real-time PCR [54]. Functionally, *hsa-miR-223-3p* modulates inflammatory responses by modulating the NF-kappaB pathway [55].

The remaining miRNAs identified in our study have so far not been mentioned in the context of MS, and their functions are poorly understood. miRNAs that have been repeatedly described to be differentially expressed in MS, e.g., *hsa-miR-142-3p*, *hsa-miR-146a-5p*, *hsa-miR-155-5p* and *hsa-miR-326* [22], were not contained in the filtering result, thus the therapy did not normalize their abnormal expression (*cf.* Waschbisch *et al.* [26]). Further studies are needed to decipher the immunological pathways involved, and to better understand the role of miRNA-dependent regulatory mechanisms in the immunopathogenesis of MS.

### 2.7. Interactions between Filtered MicroRNAs and mRNAs

**Figure 4:**
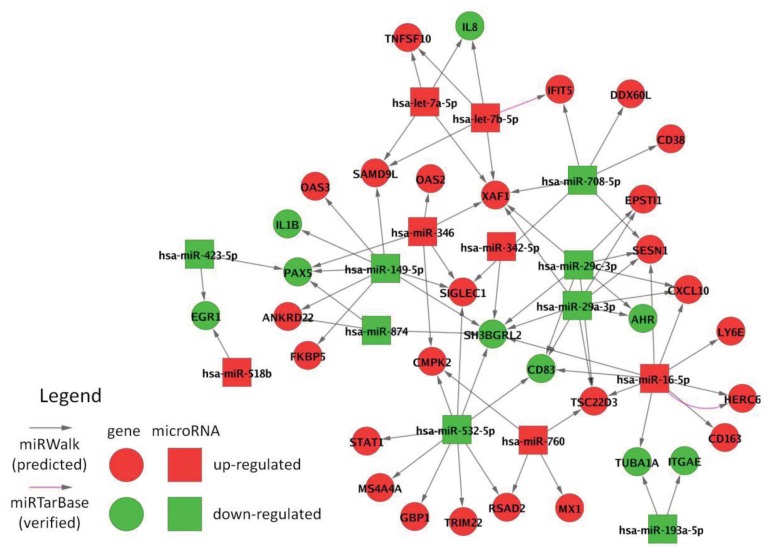
Verified and predicted interactions between IFN-beta-responsive microRNAs and mRNAs. The network of miRNA-target interactions between differentially expressed miRNAs and mRNAs was built using the databases miRWalk and miRTarBase. miRWalk reported results of 10 different prediction algorithms, and we only considered mRNA targets being computationally predicted by at least five of the 10 algorithms. miRTarBase provided experimentally validated miRNA-target interactions. In total, 15 of the filtered miRNAs were linked to 34 of the filtered genes by 74 predicted and two validated interactions. *hsa-miR-29a-3p* and *hsa-miR-29c-3p* (in the center-right) belong to the same miRNA family and are predicted to regulate eight target genes in common. The network is available as a Cytoscape session file (Supplementary File 6).

Interactions between the 20 filtered miRNAs and the 95 filtered mRNAs were derived from two databases providing potential target genes of miRNAs. The miRWalk database [56] was used to obtain miRNA-mRNA interactions consistently predicted by multiple computational algorithms. This resulted in 74 potential interactions for 15 of the 20 filtered miRNAs. The miRTarBase database [57] was used to extract interactions with experimental evidence in the literature. This resulted in two verified interactions: *hsa-miR-16-5p* was identified as a post-transcriptional regulator of HERC6 in both databases. Moreover, there is an experimentally determined interaction between *hsa-let-7b* and IFIT5. IFIT5 is a known IFN-beta-induced gene. A recent study by Abbas *et al.* characterized IFIT5 as an innate immune effector molecule acting as a sensor of viral single-stranded RNAs [58]. This confers antiviral defense by inhibiting viral replication. However, IFIT5 also recognizes cellular RNAs, including tRNAs [59]. The full network of miRNA-mRNA target interactions is visualized in Figure 4. The most connected regulators in the network were *hsa-miR-16-5p* and *hsa-miR-532-5p* with nine IFN-beta-responsive target genes each. *hsa-miR-29a-3p* and *hsa-miR-29c-3p*, which are closely related to each other, had eight predicted gene targets in common due to their similar mature sequences.

Down-regulated SH3BGRL2 and up-regulated XAF1 are simultaneously targeted by several (*n* > 5) miRNAs (miRNA target hubs [60]). Both genes have already been identified as differentially expressed in our previous studies on the effects of IFN-beta therapy [8,31] (Supplementary File 2). XAF1 is a critical mediator of IFN-beta-induced apoptosis. Its expression correlates with the cellular sensitivity to the pro-apoptotic actions of TNFSF10 [7,61]. However, while this supports the notion that miRNAs contribute to the mechanisms of action of IFN-beta, it should be noted that the majority of interactions in the network is predicted. For instance, the miRNA-mRNA interaction between *hsa-miR-16-5p* and SESN1 is predicted by nine out of 10 algorithms implemented in the miRWalk database, but has not yet been experimentally demonstrated. Another limitation is that we did not analyze whether the miRNAs bind their target mRNAs at multiple sites. Such a cooperative regulation through repetitive elements in the 3′ UTR can increase repression efficacy [62]. Additional studies are needed to validate the miRNA targets, e.g., by luciferase assays.

Opposing effects exist in the network since several mRNAs are targeted by down-regulated and up-regulated miRNAs (Figure 4). Moreover, the network does not include the effects of the transcription factors (TF), which are activated through the IFN-beta signaling pathway and which are known to regulate the expression of most of the genes [31,63]. Therefore, it is difficult to disentangle the effects of miRNA expression changes on the mRNA levels measured during therapy. Further studies are thus required, e.g., specific miRNA transfection experiments examining the impact on potential target genes at both the mRNA and protein level.

Our network analysis was limited to the set of filtered genes, and the many other potential target genes of the filtered miRNAs were out of scope. Moreover, we did not investigate whether the miRNAs target viral RNAs, which is also worth to be explored in detail [28]. Despite these limitations, we conclude that miRNA-mediated regulation plays an important role in the pleiotropic effects of IFN-beta in normal immune responses as well as in the treatment of MS.

### 2.8. Cell Type-Specific Expression of IFN-beta-Responsive MicroRNAs

**Figure 5:**
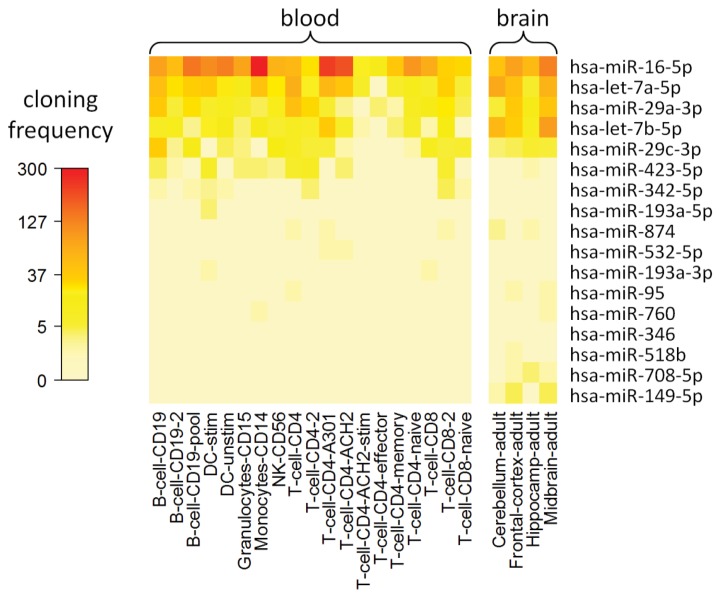
Expression of 17 filtered miRNAs in different cell populations. The heat map visualizes the levels of IFN-beta-responsive miRNAs in 19 blood cell populations and four brain tissues. The data were downloaded from the smirnaDB database [64], which did not contain three of the 20 filtered miRNAs (*hsa-miR-27a-5p*, *hsa-miR-29b-1-5p* and *hsa-miR-181c-3p*). *hsa-let-7a-5p* and *hsa-miR-16-5p* are highly expressed in peripheral blood and brain. *hsa-let-7b-5p* and *hsa-miR-149-5p* are preferentially expressed in brain tissues. Several of the miRNAs (e.g., *hsa-miR-346*) were not detected in certain cell types [65].

We analyzed whether the miRNA expression changes during therapy affect the gene expression in different cell populations involved in the disease. This was done by comparing the cell type-specific expression of the miRNAs using information from the smirnaDB database [64] (Figure 5). Of the filtered miRNAs, *hsa-miR-16-5p* is highly expressed among diverse blood cells, in particular monocytes and CD4+ T cell lines, whereas *hsa-miR-149-5p* is only detected in brain (Figure 5). Accordingly, in our time course data set, *hsa-miR-16-5p* was expressed at very high levels, and *hsa-miR-149-5p* was expressed at relatively low levels (Supplementary File 3). Several miRNAs, however, were not detected in some cell populations (clone count = 0) due to the limited sensitivity of the measurement technique used to generate these data [65]. More sensitive methods could be used to further analyze the cell type-specific roles of selected miRNAs in MS and therapy, e.g., *hsa-miR-29a-3p* and *hsa-miR-29c-3p*, which were expressed in B cells.

### 2.9. Final Remarks and Perspectives

The cellular regulation of the miRNAs is still not well understood. Many type I IFN-responsive genes harbor in their promoter region a specific sequence motif, the IFN-stimulated response element (ISRE), which is bound by IFN-activated TFs [7]. However, when we searched miRGen 2.0, a database of TF binding sites for miRNA transcripts [66], there was only one miRNA (*hsa-mir-203a*) that was predicted to have an ISRE located near the transcription start site. It is conceivable that some of the filtered miRNAs are regulated at the RNA processing level rather than at the transcriptional level. Several post-transcriptional mechanisms can affect mature miRNA biogenesis and stability [67]. Recent studies provided evidence that miRNAs can be suppressed by circular RNAs, which act as natural miRNA sponges [68]. On the other hand, the localization of the miRNAs might be altered, e.g., by microparticle shedding [69]. Additional research is needed to elucidate how the activity of miRNAs might be modulated during therapy.

Currently, there is a clear lack of studies investigating the changes in miRNA expression during MS therapy. Apart from the study by Waschbisch *et al.* [26] (see Section 1), there is only another study by Sievers *et al.*, who found differentially expressed miRNAs in B cells of patients treated with natalizumab [41]. Future miRNA profiling analyses should use a longitudinal design and address both the short-term and the long-term effects of the available treatments. Such studies may also help to understand why some patients continue to have clinical relapses, disability progression or active lesions despite therapy. Defining the individual response to treatment is difficult, but miRNAs may have the potential to be used as prognostic biomarkers, thereby facilitating improved patient care. The identification of miRNA biomarkers should be supported by functional studies on how miRNAs affect complex biological processes by targeting multiple genes in different cell types.

## 3. Experimental Section

### 3.1. Samples

Fifteen milliliters peripheral venous EDTA blood samples were taken from six patients (Table 1) immediately before first (baseline), second, and third IFN-beta injection as well as after one month. For validation, further blood samples were taken from an independent cohort of 12 patients (Supplementary File 4) before and one month after the start of IFN-beta-1b sc. therapy. The samples were always processed within one hour. PBMC were separated by isopycnic centrifugation in Ficoll density gradients, and total RNA enriched with small RNAs was isolated using the mirVana miRNA isolation kit (Invitrogen, Carlsbad, CA, USA) according to the manufacturers’ protocols. The study was approved by the University of Rostock’s ethics committee and carried out according to the Declaration of Helsinki. Written informed consent was obtained from all patients before study onset.

### 3.2. Gene Expression Profiling Using Microarrays

To quantify the mRNA levels, total RNA from each of the 24 PBMC samples of the main study cohort was labeled and hybridized to Affymetrix microarrays. Biotinylated cRNA were prepared according to the standard Affymetrix 3′ IVT protocol from 200 ng total RNA (Expression Analysis Technical Manual; Affymetrix, Santa Clara, CA, USA). Following fragmentation, 15 μg of cRNA were hybridized for 16 h at 45 °C on Affymetrix GeneChip Human Genome U133 Plus 2.0 Arrays. The microarrays were washed and stained in the Affymetrix Fluidics Station 450, and scanned using the Affymetrix GeneChip Scanner 3000 (Affymetrix, Santa Clara, CA, USA).

### 3.3. MicroRNA Expression Analysis Using Real-Time PCR

To quantify the miRNA expression levels, we used the TaqMan Array Human MicroRNA A + B Cards Set v2.0 (Applied Biosystems, Foster City, CA, USA), which consists of two 384-well plates with TaqMan assay reagents. These plates contain 720 assays to measure 651 different human miRNAs. Moreover, there are 30 assays for positive controls, 2 assays for negative controls, and 16 assays were discarded as they link to dead miRNA entries in the miRBase database (release 17) [17]. Total RNA (120 ng) from each sample (*n* = 24) was reverse transcribed to cDNA using Megaplex RT Primers in combination with Megaplex PreAmp Primers (Life Technologies, Carlsbad, CA, USA). The real-time PCR measurements were then performed with predesigned primers and TaqMan probes with 45 cycles according to the manufacturer’s instructions in a 7900HT Sequence Detection System (Applied Biosystems, Foster City, CA, USA). Raw Ct values were computed using the SDS 2.3 and RQ Manager 1.2 software (Applied Biosystems, Foster City, CA, USA), and undetermined data were set to Ct = 45.

### 3.4. Validation MicroRNA Analysis Using Microarrays

For validation, we replicated the miRNA expression measurements of the PBMC from three patients (Pat1-3) at two time points (before the first IFN-beta injection as well as after one month). Total RNA of these six samples was labeled and hybridized to Affymetrix GeneChip miRNA 2.0 arrays. Biotinylated RNA was prepared using the FlashTag Biotin HSR RNA labeling kit according to the standard Affymetrix protocol from 600 ng total RNA (Expression Analysis Technical Manual; Affymetrix). Following fragmentation, the biotin-labeled RNA was hybridized for 16 h at 45 °C on Affymetrix miRNA 2.0 arrays. The microarrays were washed and stained in the Affymetrix Fluidics Station 450, and scanned using the Affymetrix GeneChip Scanner 3000.

### 3.5. Validation MicroRNA Analysis Using Real-Time PCR

To verify the results in an independent cohort of patients, we measured 5 of the 20 filtered miRNAs in PBMC samples from 12 additional patients (Supplementary File 5). The blood samples were obtained before and after one month of IFN-beta-1b treatment. The miRNAs were selected based on a combination of different criteria, e.g., change of expression after one month according to both the TaqMan miRNA array data and the Affymetrix miRNA microarray data. The validation experiment was performed using TaqMan single-tube assays for *hsa-miR-16-5p* (Assay ID 000391), *hsa-miR-29a-3p* (Assay ID 002112), *hsa-miR-29c-3p* (Assay ID 000587), *hsa-miR-149-5p* (Assay ID 002255), and *hsa-miR-532-5p* (Assay ID 001518). Additionally, the housekeeping miRNA *hsa-miR-191-5p* (Assay ID 002299) was measured for normalization [33]. For each assay, 10 ng of total RNA from each sample (*n* = 24) were used to convert an individual miRNA to cDNA using an RT primer specific for the miRNA of interest (Applied Biosystems). The real-time PCR quantitation was performed in triplicates with predesigned primers and TaqMan probes according to the TaqMan MicroRNA Assay protocol with 45 cycles in a 7900HT Sequence Detection System (Applied Biosystems). An equivalent of 0.5 ng total RNA was used to obtain a single data point. Raw Ct values were computed using the SDS 2.3 and RQ Manager 1.2 software (Applied Biosystems).

### 3.6. Expression Data Preprocessing

In the case of the Affymetrix U133 Plus 2.0 gene expression microarrays, the raw probe-level signals were converted to expression values (signal intensities) using the MAS5.0 algorithm and custom GeneAnnot-based chip definition files (version 2.2.0) [70]. Data normalization was performed by loess normalization using the R package “affy”. Each Affymetrix GeneChip yielded mRNA levels of 19,204 human genes.

In case of the TaqMan miRNA cards, we first set the detection limit at Ct = 38 [71], and converted the raw Ct values to the linear scale using the equation 2^−Ct^ × 10^−9^. After this step, a Ct value of 38 corresponds to an expression signal of 0.004, and a Ct value of 20 corresponds to an expression signal of 953. Systematic differences in the time course data were then corrected by loess normalization. This was done separately for each patient (*n* = 6) and each card (A and B). Finally, to reduce variation in the expression signals between the patients, we scaled the data of card A and of card B so that the respective 95% quantile was the same for each patient.

In case of the Affymetrix miRNA microarrays, the raw signals were converted to expression values using the RMA algorithm with quantile normalization. For each chip, this resulted in log-transformed expression levels for 20,706 probe sets interrogating small non-coding RNA transcripts. For each of the 20 filtered miRNAs (Table 3), there was one designated probe set.

In case of the TaqMan single-tube real-time PCR miRNA assays, we set the detection limit at Ct = 38 [71], calculated the mean Ct value of each triplicate, converted the raw Ct values to the linear scale using the equation 2^−Ct^, normalized the results to the expression values of the housekeeping miRNA *hsa-miR-191-5p* [33], and scaled these ratios by a factor of 1000 for convenience.

The non-normalized and the normalized expression data of the six patients receiving IFN-beta therapy are available in the Gene Expression Omnibus (GEO) database via the SuperSeries record GSE46293. This GEO entry links to all data from the Affymetrix U133 Plus 2.0 microarrays, the TaqMan MicroRNA Cards Sets v2.0, and the Affymetrix miRNA 2.0 microarrays.

### 3.7. Filtering of Differentially Expressed mRNAs and MicroRNAs

We filtered the Affymetrix U133 Plus 2.0 and TaqMan miRNA cards data sets for IFN-beta-responsive genes and miRNAs by comparing the PBMC expression levels immediately before treatment initiation (baseline) with the expression levels two days, four days, and one month after the start of IFN-beta therapy. Up-regulation and down-regulation of genes and miRNAs were quantified using signal intensity-dependent fold-changes (MAID-scores) as described in our previous studies [8,34] (see also http://www.hki-jena.de/index.php/0/2/490). MAID-scores represent adjusted fold-changes, where a higher fold-change (*i.e.*, relative change in expression) is required for genes expressed at low levels than for genes expressed at high levels. This is realized by an exponential function (MAID regression curve) that is fitted to the signal intensity-dependent variation in the data. For each time point comparison and each type of array, we computed the MAID-score for all patients and for all measured genes and miRNAs. We then selected the genes and miRNAs being up-regulated (MAID-score above the cutoff *C*) or being down-regulated (MAID-score < −*C*) in at least four of the six patients. We chose *C* = 2 for the gene expression data set, and *C* = 1 for the miRNA expression data set. For the latter, a lower MAID-score cutoff was chosen because the larger variation in the miRNA data already leads to a higher MAID regression curve.

To provide an estimate of the number of genes and miRNAs passing the MAID filtering by chance, a permutation test was performed. The data sets were permuted 1000 times by randomly rearranging the temporal sequence of the data of each patient. The same filtering criteria as described above were then applied to each permutation.

As an alternative filtering criterion, we statistically compared the PBMC expression levels of mRNAs and miRNAs before and during treatment using paired *t*-tests (Supplementary File 2 and Supplementary File 3). However, considering the relatively small number of patients in our study, the MAID filtering method is thought to be more robust to the variation in the data.

### 3.8. Visualization of the mRNA Expression Data

A heat map was used to visualize the expression changes of the 95 filtered mRNAs (Figure 1). The heat map displays the respective data of all 6 patients before the start of therapy and after one month. To reorder the rows and columns of the data matrix, hierarchical clustering was performed with the single linkage method and Pearson’s correlation coefficient as a measure of similarity. For visualization purposes, the expression values (signal intensities) were centered and scaled row-wise (resulting in z-scores) with the standard R heat map function.

### 3.9. Evaluation of the MicroRNA Validation Data

Spearman’s rank correlation coefficient (rho) was calculated to evaluate whether the TaqMan miRNA array data correlate with the Affymetrix miRNA microarray data (120 data pairs: 20 miRNAs, three patients, and two time points). Paired *t*-tests were computed for assessing the difference in expression after one month of IFN-beta-1b sc. therapy versus baseline in the preprocessed and normalized data of the Affymetrix microarrays (Pat1-3) and of the TaqMan single-tube assays (*n* = 12 additional patients).

### 3.10. Interaction Network Analysis

We studied the regulatory interactions between the miRNAs and their target mRNAs by integrating the information from two different databases. Experimentally verified and computationally predicted target genes of the 20 IFN-beta-responsive miRNAs were extracted from the databases miRTarBase (version 3.5) [57] and miRWalk (April 2013) [56], respectively. miRWalk contained predictions for all 20 filtered miRNAs, and miRTarBase contained interactions for 9 of the 20 miRNAs. For the prediction of targets with miRWalk, we applied the option of the web server to run the calculations with 10 different prediction algorithms on 3′ UTRs of all human genes, and then gathered only the miRNA-mRNA interactions that were predicted by at least 5 of the 10 algorithms. Finally, interactions being associated with the 95 filtered genes were visualized as a network using the Cytoscape software (version 2.8.0) [72].

### 3.11. MicroRNA Expression in Different Cell Populations

To investigate the expression of the filtered miRNAs in different peripheral blood cell types and brain regions, we used the smirnaDB database, which provides expression levels of 692 human miRNAs for 170 cell populations and tissues [64]. This miRNA expression atlas is based on sequence analysis of small RNA clone libraries [65]. The relative cloning frequencies of miRNAs represent a measure of miRNA expression. However, in this data set, many miRNAs were identified at very low clone counts (*cf.* Landgraf *et al.* [65]). The data for 19 blood cell types (including three CD4+ T cell lines) and four brain tissues were downloaded from smirnaDB and visualized as a heat map in the R software environment.

## 4. Conclusions

To our knowledge, this is the first longitudinal genome-wide study examining the *in vivo* effects of IFN-beta treatment on miRNA expression in blood cells of patients with CIS or RRMS. The strongest changes in mRNA and miRNA expression were detected one month after the start of IFN-beta-1b sc. treatment. We observed that the induction of IFN-beta-responsive genes is paralleled by a preferential down-regulation of miRNAs. This suggests that the regulation of miRNAs contributes to the molecular mechanisms of action of IFN-beta in protective immune responses as well as in MS therapy. We confirmed the down-regulation of *hsa-miR-29a-3p*, *hsa-miR-29c-3p*, and *hsa-miR-532-5p* in an independent cohort of patients. We further analyzed the interactions between differentially expressed miRNAs and mRNAs. The largest number of predicted interactions to IFN-responsive genes was found for *hsa-miR-532-5p* and *hsa-miR-16-5p*. Up-regulated *hsa-miR-16-5p* was expressed at very high levels in different cell types of the blood, in particular monocytes. However, unraveling the complex gene regulatory interactions between TFs, miRNAs and genes remains a big challenge for the future. Functionally, some of the 20 filtered miRNAs (e.g., members of the mir-29 family) are associated with apoptosis and are involved in IFN signaling feedback loops. miRNA expression profiles in blood cells may provide biomarkers for monitoring the biological response to therapy to predict individual disease activity and progression. They may also help to better understand the pathogenetic mechanisms and to optimize the treatment of MS. Our results provide a rationale for subsequent studies in larger MS cohorts.

## Supplementary Information

*Supplementary File 1* (TIFF image): Distribution of the raw Ct values measured with the TaqMan miRNA arrays. The data of the A-cards (A) and the B-cards (B) are shown for all six patients and all four time points. The detection limit was set at Ct = 38.

*Supplementary File 2* (XLS Excel spreadsheet): mRNA filtering result. This table provides the 95 genes that were up-regulated or down-regulated in the PBMC of the patients two days, four days or one month after the initiation of subcutaneous IFN-beta-1b therapy. Different types of information are given for each gene, e.g., gene symbol, official full name, Entrez Gene identifier, and the MAID filtering results.

*Supplementary File 3* (XLS Excel spreadsheet): microRNA filtering result and validation. In comparison to pre-treatment levels, 20 miRNAs were found to be expressed at higher or lower levels in PBMC during IFN-beta treatment. The table provides, e.g., the TaqMan Detector identifiers, the MAID filtering outputs as well as the results from the validation analyses.

*Supplementary File 4* (XLS Excel spreadsheet): Clinical data and demographic data of the patients in the validation cohort. Twelve patients were recruited to confirm the observed expression changes of five selected miRNAs within the first month of IFN-beta-1b sc. treatment.

*Supplementary File 5* (XLS Excel spreadsheet): Validation real-time PCR data set. TaqMan single-tube assays were used to quantify the expression of five selected miRNAs before the start of IFN-beta therapy and after one month in PBMC of an independent cohort of 12 patients (Supplementary File 4). This table contains the raw Ct values of these five miRNAs and the housekeeping miRNA *hsa-miR-191-5p* [33]. The measurements were done in triplicates.

*Supplementary File 6* (CYS Cytoscape session file): miRNA-mRNA interaction network. This Cytoscape file (http://www.cytoscape.org) [72] contains computationally predicted and experimentally determined interactions between IFN-beta-responsive miRNAs and mRNAs. The interactions were obtained from the databases miRWalk [56] and miRTarBase [57]. A visualization of the network is shown in Figure 4.

## Figures and Tables

**Table 1:** Clinical data and demographic data of the patients.

Patient	Type	Age	Disease duration	EDSS (baseline)	EDSS (1 year)	Relapses (1 year)
Pat1	CIS	28	1	1.0	1.0	0
Pat2	RRMS	38	2	1.5	1.5	0
Pat3	RRMS	22	1	1.5	1.0	0
Pat4	RRMS	50	12	2.5	2.5	0
Pat5	CIS	60	2	1.5	2.5	0
Pat6	RRMS	27	2	2.0	1.0	2

Six female patients were recruited for the main cohort of this study. They were diagnosed with relapsing-remitting MS (RRMS) or clinically isolated syndrome (CIS) suggestive of MS. The age at study onset (in years) and the duration from the diagnosis to the start of IFN-beta-1b sc. therapy (in months) are shown. Additionally, the EDSS scores before treatment initiation (baseline) and after one year, as well as the number of relapses during the first year of clinical follow-up are given in the table.

**Table 2:** Numbers of microRNAs detected in the samples with the TaqMan cards set.

Patient	Baseline	~48 h	~96 h	1 month
Pat1	380	374	364	373
Pat2	375	407	392	362
Pat3	427	400	408	407
Pat4	390	387	388	389
Pat5	285	258	266	262
Pat6	431	451	446	391

Approximately 400 miRNAs were found to be expressed (raw Ct value < 38) per sample. The numbers are lower for the PBMC samples of patient Pat5, where generally lower miRNA amounts were measured with the B-cards for all four time points (Supplementary File 1).

**Table 3:** Details of microRNAs differentially expressed during IFN-beta therapy.

Mature miRNA	Sequence	Expression change	Family	pre-miRNA	pri-miRNA	Location
*hsa-let-7a-5p*	UGAGGUAGUAGGUUGUAUAGUU	up-regulated	let-7	MIRLET7A1		chr9 (q22.32)
				MIRLET7A2	MIR100HG	chr11 (q24.1)
				MIRLET7A3	MIRLET7BHG	chr22 (q13.31)
*hsa-let-7b-5p*	UGAGGUAGUAGGUUGUGUGGUU	up-regulated	let-7	MIRLET7B	MIRLET7BHG	chr22 (q13.31)
*hsa-miR-16-5p*	UAGCAGCACGUAAAUAUUGGCG	up-regulated	mir-15	MIR16-1	DLEU2	chr13 (q14.2)
				MIR16-2	SMC4	chr3 (q25.33)
*hsa-miR-27a-5p*	AGGGCUUAGCUGCUUGUGAGCA	down-regulated	mir-27	MIR27A		chr19 (p13.13)
*hsa-miR-29a-3p*	UAGCACCAUCUGAAAUCGGUUA	down-regulated	mir-29	MIR29A		chr7 (q32.3)
*hsa-miR-29b-1-5p*	GCUGGUUUCAUAUGGUGGUUUAGA	down-regulated	mir-29	MIR29B1		chr7 (q32.3)
*hsa-miR-29c-3p*	UAGCACCAUUUGAAAUCGGUUA	down-regulated	mir-29	MIR29C		chr1 (q32.2)
*hsa-miR-95*	UUCAACGGGUAUUUAUUGAGCA	down-regulated	mir-95	MIR95	ABLIM2	chr4 (p16.1)
*hsa-miR-149-5p*	UCUGGCUCCGUGUCUUCACUCCC	down-regulated	mir-149	MIR149	GPC1	chr2 (q37.3)
*hsa-miR-181c-3p*	AACCAUCGACCGUUGAGUGGAC	down-regulated	mir-181	MIR181C		chr19 (p13.13)
*hsa-miR-193a-3p*	AACUGGCCUACAAAGUCCCAGU	down-regulated	mir-193	MIR193A		chr17 (q11.2)
*hsa-miR-193a-5p*	UGGGUCUUUGCGGGCGAGAUGA	down-regulated	mir-193	MIR193A		chr17 (q11.2)
*hsa-miR-342-5p*	AGGGGUGCUAUCUGUGAUUGA	up-regulated	mir-342	MIR342	EVL	chr14 (q32.2)
*hsa-miR-346*	UGUCUGCCCGCAUGCCUGCCUCU	up-regulated	mir-346	MIR346	GRID1	chr10 (q23.2)
*hsa-miR-423-5p*	UGAGGGGCAGAGAGCGAGACUUU	down-regulated	mir-423	MIR423	NSRP1	chr17 (q11.2)
*hsa-miR-518b*	CAAAGCGCUCCCCUUUAGAGGU	up-regulated	mir-515	MIR518B		chr19 (q13.42)
*hsa-miR-532-5p*	CAUGCCUUGAGUGUAGGACCGU	down-regulated	mir-188	MIR532	CLCN5	chrX (p11.23)
*hsa-miR-708-5p*	AAGGAGCUUACAAUCUAGCUGGG	down-regulated	mir-708	MIR708	TENM4	chr11 (q14.1)
*hsa-miR-760*	CGGCUCUGGGUCUGUGGGGA	up-regulated	mir-760	MIR760		chr1 (p22.1)
*hsa-miR-874*	CUGCCCUGGCCCGAGGGACCGA	down-regulated	mir-874	MIR874	KLHL3	chr5 (q31.2)

The table lists the 20 miRNAs found to be expressed at higher or lower levels in the PBMC of patients with CIS or MS in response to IFN-beta therapy. The base sequence, the gene regulatory effect of the treatment (“Expression change”), the miRNA family, the HGNC symbols of the precursor and primary miRNAs, as well as the genomic location are shown. Two of the mature miRNAs (*hsa-let-7a-5p* and *hsa-miR-16-5p*) are processed from more than one precursor miRNA. For 11 miRNAs the pri-miRNA transcript has been annotated. None of these pri-miRNAs appeared in the mRNA filtering result. Precursor miRNAs of *hsa-let-7a-5p* and *hsa-let-7b-5p*, and of *hsa-miR-29a-3p* and *hsa-miR-29b-1-5p* are clustered, *i.e.*, they share their transcription locus.

## Supplementary Files

Supplementary File 1 Supplementary1 (TIF, 351 KB)

Supplementary File 2 Supplementary2 (XLS, 56 KB)

Supplementary File 3 Supplementary3 (XLS, 35 KB)

Supplementary File 4 Supplementary4 (XLS, 25 KB)

Supplementary File 5 Supplementary5 (XLS, 55 KB)

Supplementary File 6 Supplementary6 (CYS, 20 KB)
