# Supplementary figures and images for: MicroRNA Expression Changes during Interferon-Beta Treatment in the Peripheral Blood of Multiple Sclerosis Patients
Source: Int J Mol Sci. 2013 Aug 5;14(8):16087–110. doi: 10.3390/ijms140816087 (PMC3759901; doi:10.3390/ijms140816087)

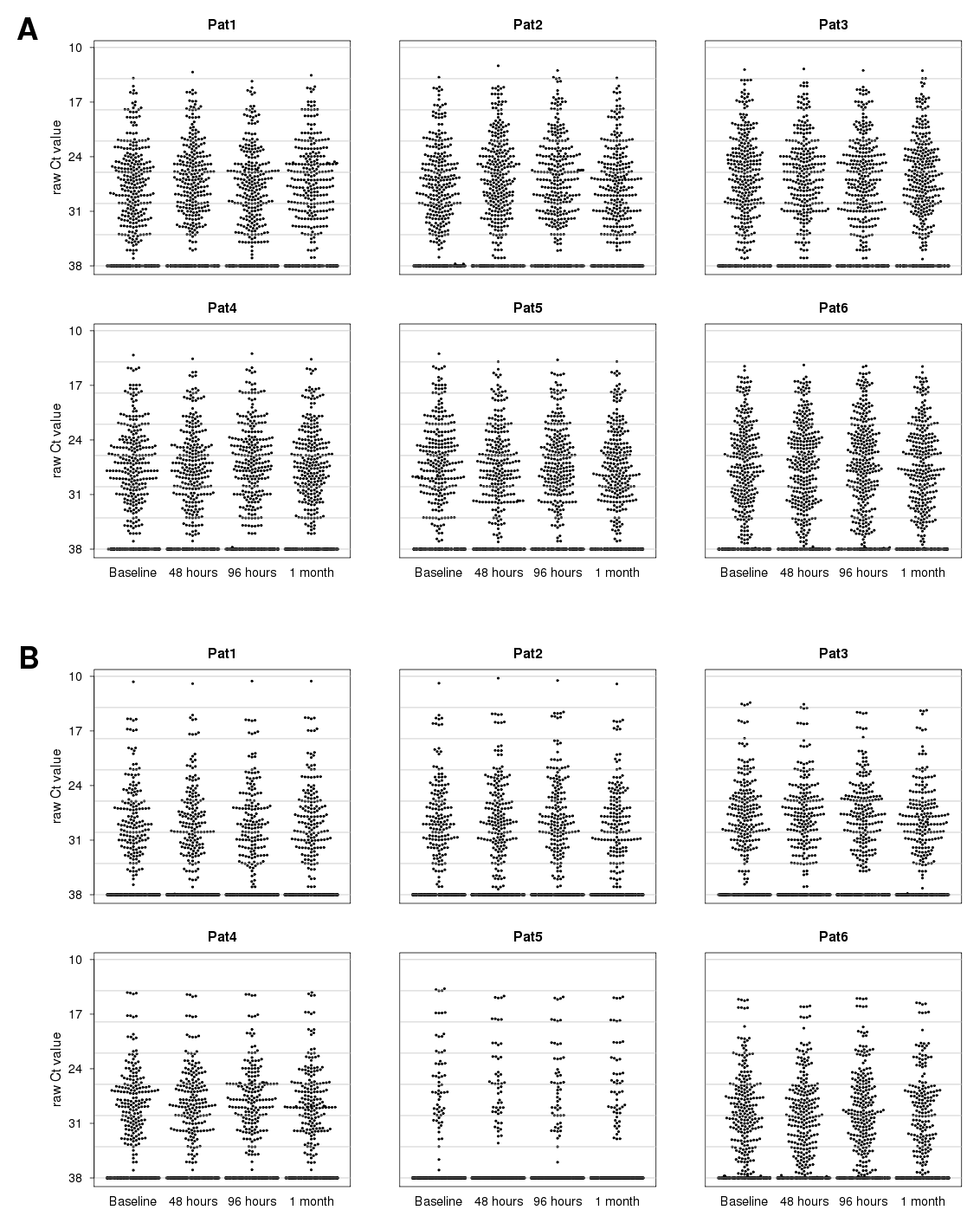

Supplement: Supplementary File 1 — Supplementary1 (TIF, 351 KB) [file ijms-14-16087-s001.tif]
